# Availability of age-appropriate paediatric formulations in the Netherlands: the need in daily clinical practice remains

**DOI:** 10.1136/ejhpharm-2019-001977

**Published:** 2019-11-11

**Authors:** Anna van der Vossen, Sandra Buljaç, Kadir Akçay, Jan Dietert Brugma, Arnold Vulto, Lidwien Hanff

**Affiliations:** 1 Department of Hospital Pharmacy, Erasmus Medical Center, Rotterdam, The Netherlands; 2 Department of Outpatient Pharmacy, Erasmus Medical Center, Rotterdam, The Netherlands; 3 Department of Hospital Pharmacy, Princess Maxima Center for Pediatric Oncology, Utrecht, The Netherlands

**Keywords:** paediatric formulations, compounding, excipients, availability

## Abstract

**Objectives:**

To quantify the availability of authorised, age-appropriate paediatric medicines in clinical practice in the Netherlands and to identify gaps by assessing dispensing practice in a paediatric hospital.

**Methods:**

The availability of age-appropriate formulations was assessed by conducting a survey on the use of pharmacy compounded medicines among the paediatric hospitals in the Netherlands, and by analysing dispensing data of oral medication from the inpatient pharmacy of the largest paediatric hospital in the Netherlands. The age-appropriateness of the dispensed formulations was assessed on two aspects: dose-capability and acceptability. Liquid drug products that are unsuitable due to the presence of potentially harmful excipients, were identified based on the dosage in clinical practice.

**Results:**

For 129 out of 139 drug substances included in the survey (93%), at least one of the eight respondents stated to use a pharmacy compounded product to meet the needs of their paediatric patients. The age-appropriateness of medicines dispensed from the inpatient pharmacy increased with age, and was higher for non-intensive care unit (ICU) patients than for ICU patients. We identified 15 drug products causing excipient exposure above the European Medicines Agency-recommended values.

**Conclusions:**

This study confirms there is still a large need for age-appropriate formulations in daily clinical practice. Pharmacy compounding for paediatric patients remains essential for many indications. The need for potentially harmful excipients in compounded products should be critically assessed.

## Introduction

Drug development for children has long been a neglected area compared with adult drug development. Low prevalence of disease and the resulting low return on investment, together with ethical and practical barriers, have not been providing enough incentives for pharmaceutical corporations to invest time and resources into bringing appropriately tested paediatric medicines to the market. It was recognised that specific legislation was needed to address this issue. Following the example of the US Best Pharmaceuticals for Children Act, the EU Paediatric Regulation (EC)^1^ No 1901/2006 was adopted in December 2006.^1^

Since the introduction of the Paediatric Regulation, many initiatives have been taken to improve the availability of paediatric drug formulations. New dosage forms such as dispersible films and multiparticulates (eg, sprinkles, mini-tablets) have been developed,^2^ and during the years 2007–2016, over 260 new medicines have been authorised in the EU for use in children, which is regarded as the success of the Paediatric Regulation.^3^ Unfortunately, we also see that the paediatric use marketing authorisation (PUMA) delivered a very limited number of age-appropriate paediatric formulations for off-patent drugs, with only four PUMAs granted during the first 10 years of the Paediatric Regulation.^4^

Looking at the European Medicines Agency (EMA) priority list of off-patent medicinal products,^5^ and the inventory of needs for paediatric medicines,^6^ a discrepancy emerges between the availability of marketed paediatric medicines and the medicines needed in daily practice. Within the Netherlands, the limited commercial availability of authorised medicines for children has previously been recognised by van Riet-Nales *et al*.^7^ These authors compared dosing information for use in children from a national Medicines Compendium (Informatorium Medicamentorum) with the official indications in the Summary of Product Characteristics (SmPC), and found a 48% overall availability of authorised medicines for children. Furthermore, the age-appropriateness of the formulation, as well as the presence of potentially harmful excipients were assessed, confirming a lag in pharmacotherapeutic treatment options compared with adults. However, this study did not involve the need in clinical practice in its design.

The absence of age-appropriate, authorised and commercially available dosage forms is forcing pharmacists to compound drugs, or caregivers to manipulate adult formulations before administration. Individual compounding and manipulation of medicines can be costly and time-consuming, but most importantly carry risks for the patient. Examples of safety issues linked to compounding include decreased bioavailability of a tacrolimus suspension,^8^ and a 10-fold dosing error of spironolactone due to the availability of different strengths.^9^ Manipulations such as crushing of tablets can lead to loss of controlled release properties, or loss of drug substance.^10^ Furthermore, splitting of both scored and unscored tablets can lead to dose inaccuracies, and splitting devices are not necessarily more accurate than splitting by hand.^11 12^ Another important disadvantage of the use of unstandardised compounded medicines is the absence of clinical decision support with electronic prescribing.

Regardless of the authorisation status, a lot of medicines dispensed to paediatric patients are not age-appropriate, either because of unacceptability of the dosage form to the patient or because of incapability to provide the correct dose.^7^ The acceptability of different dosage forms to paediatric patients has been summarised in the ‘Reflection paper on formulations of choice for the paediatric population’ by the EMA.^13^ It provides a matrix proposing applicability and acceptability of different dosage forms in specific age groups, based on a limited amount of evidence. For this reason, it was presented as a rough guide, and not an evidence-based recommendation for the development of dosage forms. Since then, acceptability studies of different dosage forms have become available, but the methodologies have not been standardised, and for some age groups and dosage forms, no consensus has been obtained.^14^

One aspect determining the age-appropriateness of medicines is the presence of potentially harmful excipients. Excipients are generally considered to be pharmacologically inactive, but they pose a risk for patients with immature metabolic pathways and organ systems. For several of them, the EMA has published reflection papers or questions and answers, addressing the safety issues with use in paediatric medicines. These potentially harmful excipients are frequently used in liquid formulations, but their harmfulness is relative to exposure and patient characteristics. Excipient exposure in preterm infants and neonates has previously been assessed for several substances.^15–17^ These studies showed that a lot of drug products used in paediatrics are possibly unsuitable due to their excipients, but to date, this has only been evaluated for the youngest patients.

In summary, there is still a limited availability of commercial and age-appropriate paediatric medicines, but the magnitude of the problem in clinical practice has not been determined. The aim of this research was to quantify the availability of commercial, age-appropriate paediatric medicines and to identify gaps. In order to achieve this aim, we made use of different strategies and datasets and in contrast to earlier work, this study specifically focuses on daily clinical practice.

The availability of commercial drug products, restricted to oral medication, was assessed using two datasets: 1) a survey on the use of pharmacy compounded (non-commercial) medicines among the paediatric hospitals in the Netherlands and 2) dispensing data from the inpatient pharmacy of the largest paediatric hospital in the Netherlands. Subsequently, the age-appropriateness of the dispensed oral formulations was assessed according to EMA acceptability guidance and additional criteria previously applied by van Riet-Nales *et al*.^7^ Finally, we identified liquid drug products that are unsuitable due to the presence of potentially harmful excipients, based on the extent of exposure in clinical practice.

## Methods

### Availability of paediatric medicines in the Netherlands

In 2016, a survey was conducted among the 11 Dutch academic and teaching paediatric hospitals, to identify the use of pharmacy compounded medicines for paediatric patients in the Netherlands. For the survey, we established a list of drugs of interest based on the existing monographs of the Dutch Paediatric Formulary.^18 19^ Based on route of administration (oral), unavailability of a commercial oral liquid dosage form, and the absence of an equivalent therapeutic alternative (eg, pantoprazole and omeprazole), we included 139 drug substances (online supplementary appendix 1) in the survey. Respondents were asked to confirm if 1) the drug was applied for their patients, 2) a commercially available product was able to meet the needs of their patients and 3) they made use of a pharmacy compounded product. Furthermore, we asked them to supplement the list with any products they thought were missing. The results were subsequently compared with the EMA inventory of paediatric needs.

10.1136/ejhpharm-2019-001977.supp1 Supplementary data

To supplement the qualitative data collected in the survey, we used the prescription and dispensing data of the Erasmus MC-Sophia Children’s Hospital to quantify for which age groups dispensing of pharmacy compounded, non-commercial products was most prevalent. In this dataset, non-Dutch Paediatric Formulary medicines were also included. Age categories were defined according to the guideline on clinical investigation of medicinal products in the paediatric population,^20^ and all patients admitted to the neonatal intensive care unit (NICU) were categorised as preterm neonates. All electronically prescribed medication orders, for patients admitted to the paediatric intensive care unit (PICU), NICU or the remaining non-ICU units (surgical, oncology and general wards) were evaluated at start of treatment. The electronic prescription data were corrected when the dispensed dosage form deviated from the prescription. A MSc pharmacy student prospectively collected these discrepancies at the inpatient pharmacy on weekdays over a period of 10 weeks during the autumn of 2016.

### Age-appropriateness of paediatric formulations dispensed from the inpatient pharmacy

In addition to the availability of commercial drug products, we evaluated the dose-capability (the capability to deliver the correct dose) and age-appropriateness of all oral medication dispensed from the pharmacy according the criteria previously applied by van Riet-Nales *et al,* 7 using the dispensing dataset described above. In this dataset, we also included injections fluids dispensed for oral administration. To assess dose-capability, manipulations to the dispensed product required to obtain the correct dose, such as tablet splitting, were verified with the SmPC. Age-appropriateness of the formulation was determined using the acceptability matrix of the EMA reflection paper,^13^ supplemented with the criteria displayed in table 1. The EMA matrix, combining different age groups, routes of administration and dosage forms, assigns levels of either applicability (younger ages) or acceptability (higher ages) ranging from 1: not applicable/not acceptable to 5: best and preferred applicability/dosage form of choice. We considered a value of 4 or 5 to represent sufficient suitability. Different from van Riet-Nales *et al,* we considered capsules that may be opened, and tablets that may be pulverised according to the SmPC, to be suitable for children from the age of 2 years, instead of the age of 1 month.

**Table 1:** Additional suitability criteria for paediatric oral dosage forms aside from the EMA matrix^7^

Tablets	A single dose may involve two tablets at the maximum
	A single dose may involve a halved tablet, if 1) the tablet contains a score line; 2) the SmPC does not state that the scoring line is for esthetical reasons only; 3) the SPC does not state that the tablet may only be broken to facilitate the intake of the full dose.
**Oral liquid preparations**	The maximum dosing volume is 5 mL for children aged below 5 years.
	The maximum dosing is 10 mL for children aged from 5 to 10 years.
	The minimum single dosing is 0.2 mL.

SmPC, Summary of Product Characteristics.

### Excipients in paediatric formulations

To identify liquid drug products that are unsuitable due to the presence of potentially harmful excipients, four commonly used excipients with known risks were selected: ethanol, propylene glycol (PG), benzyl alcohol and propyl paraben. To allow for comparison with previously published work, non-oral formulations were also included. Cut-off values for maximum excipient exposure considered to be suitable for a certain age group were retrieved from EMA publications and are summarised in table 2. As there are no daily limits published for ethanol, we interpreted the single dose limits from the current draft EMA document on ethanol as daily limit.^21^ To quantify the exposure of our patients to potentially harmful excipients, we studied the actual dosages and drug formulations administered at the paediatric wards, also including parenteral and rectal formulations. Information on the composition of the formulations was retrieved from the SmPC or via direct communication with the marketing authorisation holder or manufacturer. The dataset for the analysis contained all ongoing medication orders for each single day in February 2017 and was obtained from the electronic prescribing systems of the Erasmus MC-Sophia Children’s Hospital. The daily administered amounts of excipients were calculated for each individual patient and compared with the recommended values for safe exposure. If patients were on multiple medicines simultaneously, this was factored into the daily exposure calculation. After identifying patients with potentially harmful exposure, we calculated the median (range) exposure per product and age group.

**Table 2:** Excipients and cut-off values for safe exposure per age group, derived from EMA publications

Excipient	Age	Limit	Explanation
Ethanol^21^	<2 years	Avoid	
	2–5 years	6 mg/kg	Suggested limit in medicines based on a BAC rise of 0.01 g/L.
	≥6 years	75 mg/kg	Suggested limit in medicines based on a BAC rise of 0.125 g/L.
Propylene glycol^26^	Neonates	1 mg/kg/day	Considered to be safe and with no noticeable effects whatever the duration and the route of administration.*
	1 month–4 years	50 mg/kg/day	
	≥5 years	500 mg/kg/day	
Benzyl alcohol^29^	Preterms and neonates	Not recommended	
Propyl paraben^30^	Any	2 mg/kg/day	Permitted daily exposure according to the method outlined in ICH Q3C.

*With the exception of inhalation.

BAC, blood alcohol concentration; EMA, European Medicines Agency; ICH Q3C, International Conference on Harmonisation of Technical Requirements for Registration of Pharmaceuticals for Human Use guideline Q3C.

### Data analysis

Descriptive statistics were performed with Microsoft Excel 2010.

## Results

### Availability of paediatric medicines in the Netherlands

Out of the 11 academic and teaching paediatric hospitals that were approached, 8 responded and filled out the questionnaire. The survey revealed that for 129 out of 139 drug substances (93%), at least one of the eight respondents stated that a compounded product was needed to meet the needs of their paediatric patient. Table 3 displays all medicines for which at least five respondents stated to use a compounded drug. For 13 of these 28 drugs (46%), the EMA inventory of paediatric needs does not state the need for an age-appropriate formulation. Importation from other countries occurred for 25 drug substances (18%), most cited were valganciclovir (four respondents), and doxapram, nitazoxanide and alimemazine (two respondents).

**Table 3:** Most frequently used compounded drugs across paediatric hospitals in the Netherlands

Drug	Therapeutic class according to EMA needs for paediatric medicines	Formulation requirement according to EMA needs for paediatric medicines
Acetazolamide	Neurology	No
Amlodipine	Nephro-urology	Yes
Caffeine	Respiratory	No
Carvedilol	Cardiovascular	Yes
Chloral hydrate	Neurology/Psychiatry	Yes
Clobazam	Neurology	No
Clonidine	Cardiovascular	Yes
Dexamethasone	Endocrinology	No
Enalapril	Nephro-urology	Yes
Furosemide	Nephro-urology	No
Hydrochlorothiazide	Nephro-urology	Yes
Hydrocortisone	Endocrinology/Immunology	Yes
Labetalol	Cardiovascular	No
Lorazepam	Neurology/Psychiatry	Yes
Methadone	Pain	No
Midazolam	Anaesthesiology/Psychiatry	Yes
Nifedipine	Nephro-urology	Yes
Pancreatine	Gastroenterology	Yes
Phenobarbital	Neurology	Yes
Phenytoin	Neurology	No
Prednisolone	Rheumatology/Immunology	Yes
Propranolol	Cardiovascular	No
Sildenafil	Cardiovascular	No
Sodium benzoate	Metabolic disorders	No
Sotalol (hydrochloride)	Cardiovascular	Yes
Spironolactone	Nephro-urology	No
Tacrolimus	Immunology	No
Topiramate	Neurology/Psychiatry	Yes

EMA, European Medicines Agency.

### Dispensing of commercial products from the inpatient pharmacy

**Figure 1:**
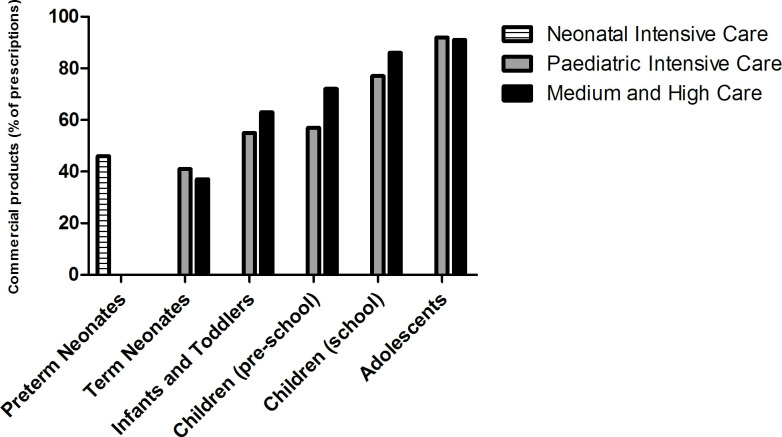
Prevalence of dispensing of commercial oral drug products per age category.

Over the 10-week study period during the autumn of 2016, 2274 oral medication orders were evaluated for a total of 437 patients. Our data show that the use of commercially available drugs was lowest in preterm neonates (193/418 prescriptions, 46%) and neonates at the PICU (33/80 prescriptions, 41%) and non-ICU wards (20/54 prescriptions, 37%). Figure 1 displays the percentage of commercial products dispensed per age group, for ICU and non-ICU patients.

### Age-appropriateness of paediatric formulations dispensed from the inpatient pharmacy

**Figure 2:**
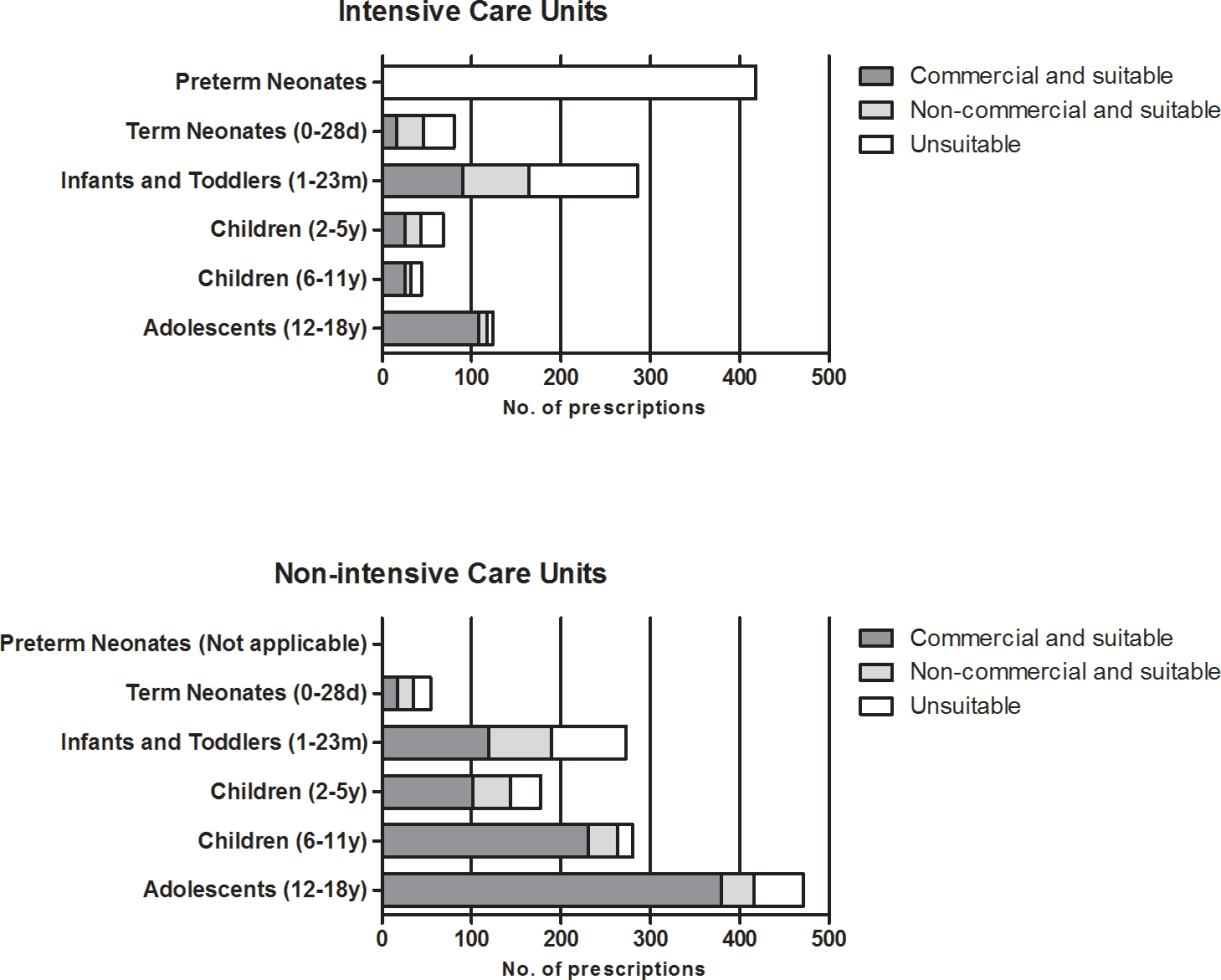
Suitability of oral dosage forms dispensed from the inpatient pharmacy.

Results from the dose-capability and age-appropriateness assessment depicted in figure 2 revealed that only 402/601 (67%) of dispensed oral formulations for the PICU were considered suitable for the patient according to the set criteria. For the non-ICU wards this number was higher, with 1047/1255 (83%) dispensed oral formulations regarded as suitable. For the NICU, all 418 dispensed oral formulations were considered unsuitable, as the EMA dosage form matrix considers all oral dosage forms to be ‘applicable with problems’ in preterm new-born infants. Outside of the NICU, dispensing of unsuitable products was most prevalent in neonates and infants at the ICU, with a percentage of 42% in both groups. This was mainly the result from dispensing of solid dosage forms, which are considered unsuitable according to the EMA matrix up to an age of 2 years. The percentage of dispensed suitable formulations increased with age, up to 94% in adolescent ICU patients and 88% in adolescent non-ICU patients.

### Excipients in paediatric formulations

For the identification of unsuitable drug products due to the presence of potentially harmful excipients, we used a second dataset with prescription data from the inpatient wards of the Sophia Children’s Hospital from February 2017. A total of 383 unique patients were admitted and received medication during the study period. From a total of 14 449 medication orders, we identified 40 drug products containing the selected excipients. The cut-off values for excipient exposure were surpassed in 22/33 (67%) of NICU patients, 18/77 (23%) of PICU patients and 16/311 (5%) of non-ICU patients. Exposure sometimes continued over multiple days (median 6, range 1–15 days), and was most frequent with the use of caffeine oral liquid (16 patients, PG), nystatin suspension (10 patients, ethanol) and alprostadil infusion (9 patients, ethanol), which are all administered for prolonged periods if necessary. For PG, the highest daily exposure was observed for diclofenac intravenous, lorazepam intravenous and itraconazole oral liquid.

In total, we identified 15 products that caused excipient exposure above the recommended values, as displayed in table 4. Five of these products were pharmacy compounded, non-commercial liquids. Propranolol, furosemide and hydrochlorothiazide liquids were prepared according to the Formulary of Dutch Pharmacists.^22^ PG in these products comes from a concentrated methyl paraben solution (15% m/v), used to process the preservative. No benzyl alcohol administration above cut-off value was observed during our study period.

**Table 4:** Drug products causing excipient exposure above the cut-off values, the number of patients exposed and the corresponding median (range) daily exposure per product and age group

Generic drug name	Brand drug name	Route of administration	Drug concentration	Ethanol concentration (mg/mL)	Age group	No. of patients	Ethanol (mg/kg/day) Median	Range
Alprostadil	Prostin VR	Intravenous	0.5 mg/mL	790	Neonates	7	18.7	14.0–80.6
					Infants and toddlers	2	64.8	14.4–144
Amphotericin B	Fungizone	Per os	100 mg/mL	4.21	Neonates	3	0.9	0.8–1.0
					Infants and toddlers	6	0.6	0.4–2.1
Clemastine	Tavegyl	Intravenous	1 mg/mL	70	Infants and toddlers	3	6.3	6.2–6.4
Diazepam	Rectiole	Rectal	2 mg/mL	100	Infants and toddlers	2	1.8	1.8–1.9
Digoxin	Lanoxin PG Elixer	Per os	0.05 mg/mL	81.7	Infants and toddlers	1	5.4	
Nystatin	Labaz	Per os	100 000 U/mL	7.9	Preterms	7	23.8	9.7–48.9
					Infants and toddlers	3	7.8	1.9–11.8
				**Propylene glycol concentration (mg/mL)**			**Propylene glycol (mg/kg/day) Median**	
Caffeine	Non-commercial liquid	Per os	10 mg/mL	9.1	Preterms	16	4.8	4.3–9.2
					Neonates	4	4.6	3.3–9.5
Diclofenac	Generic	Intravenous	25 mg/mL	200	Infants and toddlers	3	72.8	70.6–81.0
Furosemide	Non-commercial liquid	Per os	2 mg/mL	9.1	Preterms	4	4.8	3.9–5.6
					Neonates	7	13.1	5.7–26.4
Hydrochlorothiazide	Non-commercial liquid	Per os	0.5 mg/mL	9.1	Preterms	4	8.0	6.9–16.4
Itraconazole	Trisporal liquid	Per os	10 mg/mL	103.6	Children	1	52.1	
Lorazepam	Temesta	Intravenous	4 mg/mL	823	Infants and toddlers	2	82.3	64.6–123.5
Potassium chloride	Non-commercial liquid	Per os	1 mmol/mL	6.1	Neonates	1	13.3	
Propranolol	Non-commercial liquid	Per os	1 mg/mL	2.275	Preterms	1	4.4	2.5–5.1
				**Propyl paraben concentration (mg/mL)**			**Propyl paraben (mg/kg/day) Median**	
Paracetamol	DARO liquid	Per os	24 mg/mL	0.56	Infants and toddlers	3	2.1	2.0–2.1
					Children	1	2.1	2.0–2.1

## Discussion

The results from this study show that 10 years after the introduction of the Paediatric Regulation, there is still a large need for age-appropriate formulations in daily clinical practice. The largest need was observed for the youngest age groups from neonates to infants and toddlers, and the need was higher at ICU wards compared with non-ICU wards.

The widespread use of pharmacy compounded products confirms that the currently available commercial products do not meet the needs of paediatric patients. Almost half of the commonly used compounded products in the Netherlands were not included in the EMA inventory of paediatric needs. This inventory stems from a report adopted by the Paediatric Committee in December 2010, on the survey of all paediatric uses of medicines in Europe. Interestingly, for the Netherlands, only outpatient data were provided for the survey by the Paediatric Committee,^23^ which may be an explanation for the discrepancies between the inventory and our results.

Individual compounding carries risks for the patient. In the Netherlands, to mitigate these risks, the Formulary of Dutch Pharmacists aims to standardise compounding and increase the quality. This formulary contains over 160 standardised monographs for extemporaneous formulations, and for each product, quality and shelf-life data are available. Many of these unauthorised products are produced under Good Manufacturing Practice(GMP) conditions in large compounding pharmacies, to obtain medicines of high pharmaceutical quality. Within hospital affiliated GMP facilities, they can be produced at relatively low cost, as there is no profit motive. The Dutch drug laws ensure that once a commercial product is marketed, extemporaneous products can no longer be supplied to other pharmacies. Compounding of Officinal Formula, meaning any medicinal product which is prepared in a pharmacy in accordance with the prescriptions of a pharmacopoeia and is intended to be supplied directly to the patients served by the pharmacy in question, is however still allowed. On a European level, in November 2011, The European Directorate for the Quality of Medicines & HealthCare (EDQM) has commenced to generate a pan-European paediatric formulary, to improve access to suitable and age-appropriate formulations. This formulary will contain monographs of extemporaneous formulations based on the best approaches currently available in national or regional formularies within Europe.^24^

Analysis of our own dispensing data showed that (preterm) neonates and infants were most likely to receive non-commercial, compounded formulations. This can be expected as older children are more likely to be able to receive the correct dose using (manipulated) adult dosage forms. However, the dispensing of a commercial product does not mean that the dosage form is suitable for the patient. When comparing our results with the results of van Riet-Nales *et al*, who conducted their research 7 years earlier and from a regulatory perspective, the percentage of authorised and dose-capable medicines with an age-appropriate formulation was very similar. With our study, these results can now be confirmed from a clinical perspective. Unfortunately, we must conclude that progress has been limited.

In the assessment of excipient exposure from liquid products, we found that possible harmful exposure was not limited to only NICU patients, but was relevant in children up to the age of 4 years.

Whittaker *et al* 16 observed ethanol exposure in preterm infants up to 1.8 mL of ethanol per week (1422 mg), uncorrected for weight. In our NICU population ethanol exposure was mainly caused by nystatin treatment, which has a standard dosing schedule of 1 mL four times daily, leading to a cumulative exposure of 0.28 mL of ethanol per week (221 mg), which is significantly lower. A follow-up study by the same group found that ethanol concentration in neonates were not elevated after exposure through medication, but they did find elevated levels of acetaldehyde.^17^ This supports the concept that neonates have minimal systemic exposure to ethanol after enteral administration at the studied dose levels, due to a first-pass effects, but exposure to acetaldehyde might be just as relevant. At the PICU, alprostadil infusions led to ethanol exposure as high as 0.18 mL/kg/day in infants and toddlers, which is equivalent to 1 (NL) unit of alcohol for a 70 kg adult. The fact that it is administered intravenously, also means that there is no first-pass effect to decrease the systemic exposure. Currently, there is no alternative for this treatment, but fortunately the duration of therapy is limited. Even though the effect of long-term exposure to low levels of ethanol in medicines on the health and development of children has not been evaluated, there is broad consensus among medicines agencies that exposure in children should be minimised.^21^

The levels of PG exposure we observed in our population were relatively low compared with the exposure reported by Whittaker *et al.* In preterms and neonates, the WHO acceptable daily intake limit of 25 mg kg^-1^ 16 was not exceeded, but the EMA limit in neonates of 1 mg kg^-1^ day^-1^ was. In infants, toddlers and children, we identified three products that produced significant exposure; diclofenac and lorazepam intravenous fluid, and itraconazole liquid. Especially the latter is concerning, as treatment often continues over several months, and a therapeutic alternative is not available.

Compared with the results reported by Akinmboni *et al*,^25^ excipient exposure in our NICU patients was lower (67% vs 98%) compared with exposure in their study population of 106 low birth weight preterm neonates. It is notable that they observed eight different products containing benzyl alcohol, although over a study period of a full year, opposed to zero products in our 1 month study period. In total, they identified 19 products containing unwanted excipients at the NICU alone, compared with only 5 in our NICU population. This difference can be explained by substitution of unfavourable products with pharmacy compounded alternatives, free of unwanted excipients, one example being ranitidine oral liquid, which is compounded in an ethanol-free formulation with a concentration of 15 mg/mL.

Overall, excipient exposure in our patients was lower compared with other studies. This is probably the result of the awareness within pharmacy compounding for potential harmful excipients. Nevertheless, we identified non-essential products that we should either try to avoid or substitute, and essential medicines in need of improvement. On the other hand, it is important to note that the cut-off values used in this study should not be interpreted as absolute limits. It should be kept in mind that higher doses may be administered when justified.^26^ The suitability assessment in this study focused on four commonly used solvents and preservatives, but there are more excipients with reports of possible toxicity in paediatric patients, including sweeteners, solubilising agents and flavourings.^27 28^

### Strengths and limitations

The major strength of this study was the use of clinical dispensing data, which enabled the identification of relevant needs in different age groups and level of care settings. Also, we included the entire age range of paediatric patients in our research. The suitability assessment revealed that at least one-third of dispensed oral dosage forms for the PICU and one-sixth of non-ICU oral medication were not age-appropriate. These results must be interpreted with caution, as the acceptability matrix from the EMA reflection paper was based on sparse evidence. If more recent evidence on acceptability of mini-tablets and multiparticulate dosage forms would have been included in the matrix, the results might have differed slightly. Unfortunately, rather few of those kinds of dosage forms are actually available. Also, even though 3 years have passed since data collection took place, none of the most frequently compounded products presented in the results have been replaced with a commercial formulation. Other aspects that might decrease the ability of patients to take solid dosage forms, such as sedation and/or tube feeding, were not considered, but almost all of the solid dosage forms that were found suitable for children and adolescents can be administered through a feeding tube after manipulation, which means that the results would largely remain the same. Palatability, which is an important component of acceptability, was not considered in the assessment, as it is unknown for most drugs. Future research should focus on generating evidence on patient preference and acceptability of dosage forms, to further assist the development of suitable paediatric drug products. Data collection took place during a specific time of the year, which means that we could have missed some medications that are seasonally dependent.

## Conclusion

This study confirms there is still a large need for age-appropriate formulations in daily clinical practice, despite the successes of the Paediatric Regulation. Pharmacy compounding for paediatric patients remains essential for many indications, and the EDQM paediatric formulary is therefore warranted. Concomitantly, efforts should be made to reduce the exposure to potentially harmful excipients, by avoiding or substituting non-essential medicines, and improving the composition of essential medicines.

**What this paper adds**

**What is already known on this subject**

- The paediatric use marketing authorisation has delivered a very limited number of age-appropriate paediatric formulations for off-patent drugs.
- There is still a limited availability of commercial and age-appropriate paediatric medicines.

**What this study adds**

- Using a survey among paediatric hospitals in the Netherlands, we identified the most commonly compounded paediatric medicines.
- We identified liquid drug products that are unsuitable due to the presence of potentially harmful excipients by linking excipient contents to actual prescribed dosages.

## Data Availability

No data are available.

## References

[1] . Regulation (EC) no 1901/2006 of the European Parliament and of the Council of 12 December 2006 on medicinal products for paediatric use (2006).

[2] Bar-Shalom D , Rose K . Pediatric formulations: a roadmap 2016.

[3] European Commission . State of Paediatric Medicines in the EU - 10 years of the EU Paediatric Regulation. Brussels 2017 26.10.2017. Contract No.: COM, 2017

[4] European Medicines Agency with its Paediatric Committee . 10-Year report to the European Commission: general report on the experience acquired as a result of the application of the paediatric regulation. London: European medicines Agency, 2017. Available: https://ec.europa.eu/health/sites/health/files/files/paediatrics/2016_pc_report_2017/ema_10_year_report_for_consultation.pdf

[5] Paediatric Committee . Revised priority list for studies on off-patent paediatric medicinal products. London: European medicines Agency, 2013. Available: https://www.ema.europa.eu/en/documents/other/revised-priority-list-studies-patent-paediatric-medicinal-products_en.pdf

[6] European Medicines Agency . Paediatric medicines - Needs for paediatric medicines London, 2017. Available: http://www.ema.europa.eu/ema/index.jsp?curl=pages/regulation/document_listing/document_listing_000096.jsp&mid=WC0b01ac0580925b1e

[7] van Riet-Nales DA , de Jager KE , Schobben AFAM , et al . The availability and age-appropriateness of medicines authorized for children in the Netherlands. Br J Clin Pharmacol 2011;72:465–73. 10.1111/j.1365-2125.2011.03982.x 21477143 PMC3175516

[8] Reding R , Sokal E , Paul K , et al . Efficacy and pharmacokinetics of tacrolimus oral suspension in pediatric liver transplant recipients. Pediatr Transplant 2002;6:124–6. 10.1034/j.1399-3046.2002.01052.x 12000467

[9] Standing JF , Tuleu C . Paediatric formulations—Getting to the heart of the problem. Int J Pharm 2005;300:56–66. 10.1016/j.ijpharm.2005.05.006 15979830

[10] Richey RH , Hughes C , Craig JV , et al . A systematic review of the use of dosage form manipulation to obtain required doses to inform use of manipulation in paediatric practice. Int J Pharm 2017;518:155–66. 10.1016/j.ijpharm.2016.12.032 28040560

[11] van Riet-Nales DA , Doeve ME , Nicia AE , et al . The accuracy, precision and sustainability of different techniques for tablet subdivision: breaking by hand and the use of tablet splitters or a kitchen knife. Int J Pharm 2014;466:44–51. 10.1016/j.ijpharm.2014.02.031 24561329

[12] Habib WA , Alanizi AS , Abdelhamid MM , et al . Accuracy of tablet splitting: comparison study between hand splitting and tablet cutter. Saudi Pharm J 2014;22:454–9. 10.1016/j.jsps.2013.12.014 25473334 PMC4246398

[13] Committee for medicinal products for human use (CHMP). reflection paper: formulations of choice for the paediatric population. London 2006.

[14] Walsh J , Ranmal SR , Ernest TB , et al . Patient acceptability, safety and access: a balancing act for selecting age-appropriate oral dosage forms for paediatric and geriatric populations. Int J Pharm 2017. 10.1016/j.ijpharm.2017.07.017 28705619

[15] Shehab N , Lewis CL , Streetman DD , et al . Exposure to the pharmaceutical excipients benzyl alcohol and propylene glycol among critically ill neonates. Pediatric Critical Care Medicine 2009;10:256–9. 10.1097/PCC.0b013e31819a383c 19188870

[16] Whittaker A , Currie AE , Turner MA , et al . Toxic additives in medication for preterm infants. Arch Dis Child Fetal Neonatal Ed 2009;94:F236–40. 10.1136/adc.2008.146035 19158148

[17] Pandya HC , Mulla H , Hubbard M , et al . Essential medicines containing ethanol elevate blood acetaldehyde concentrations in neonates. Eur J Pediatr 2016;175:841–7. 10.1007/s00431-016-2714-x 26997167 PMC4868857

[18] Stichting Nederlands Kenniscentrum voor Farmacotherapie bij Kinderen . Kinderformularium 2016 [9 May 2016]. Available: https://www.kinderformularium.nl/

[19] van der Zanden TM , de Wildt SN , Liem Y , et al . Dutch paediatric pharmacotherapy expertise network N. developing a paediatric drug formulary for the Netherlands. Arch Dis Child 2017;102:357–61. 27799154 10.1136/archdischild-2016-311674

[20] Spoltore C . Ich topic E11. clinical investigation of medicinal Producs in the paediatric population. Document CPMP/ICH/2711/99. GIORNALE ITALIANO DI FARMACIA CLINICA 2000;14:195–201.

[21] European Medicines Agency . Questions and Answers on Ethanol in the context of the revision of the guideline on ‘Excipients in the label and package leaflet of medicinal products for human use’ (CPMP/463/00), 2014. Available: https://www.ema.europa.eu/en/documents/scientific-guideline/questions-answers-ethanol-context-revision-guideline-excipients-label-package-leaflet-medicinal_en.pdf

[22] Wetenschappelijk Instituut Nederlandse Apothekers . Formularium Der Nederlandse apothekers. Den Haag: Koninklijke Nederlandse Maatschappij ter Bevordering der Pharmacie, 2013.

[23] European Medicines Agency . Report on the survey of all paediatric uses of medicinal products in Europe, 2010. Available: https://www.ema.europa.eu/en/documents/report/report-survey-all-paediatric-uses-medicinal-products-europe_en.pdf

[24] European Directorate for the Quality of Medicines and Healthcare . European Paediatric Formulary: Background & Mission Strassbourg: Counsil of Europe, 2018. Available: https://www.edqm.eu/en/background-mission-1

[25] Akinmboni TO , Davis NL , Falck AJ , et al . Excipient exposure in very low birth weight preterm neonates. J Perinatol 2018;38:169–74. 10.1038/jp.2017.165 29095430 PMC5790602

[26] Committee for Human Medicinal Products (CHMP) . Background review for the excipient propylene glycol. London: European medicines Agency, 2014. Available: https://www.ema.europa.eu/en/documents/report/background-review-excipient-propylene-glycol-context-revision-guideline-excipients-label-package_en.pdf

[27] Ernest TB , Elder DP , Martini LG , et al . Developing paediatric medicines: identifying the needs and recognizing the challenges. J Pharm Pharmacol 2007;59:1043–55. 10.1211/jpp.59.8.0001 17725846

[28] Ursino MG , Poluzzi E , Caramella C , et al . Excipients in medicinal products used in gastroenterology as a possible cause of side effects. Regul Toxicol Pharmacol 2011;60:93–105. 10.1016/j.yrtph.2011.02.010 21354240

[29] Committee for human medicinal products (CHMP). benzyl alcohol and benzoic acid group used as excipients. London 2017.

[30] Committee for Medicinal Products for Human Use . Reflection paper on the use of methyl- and propylparaben as excipients in human medicinal products for oral use. in: agency em, editor. London, 2015. Available: https://www.ema.europa.eu/en/documents/scientific-guideline/reflection-paper-use-methyl-propylparaben-excipients-human-medicinal-products-oral-use_en.pdf

