# Supplementary material for: Availability of age-appropriate paediatric formulations in the Netherlands: the need in daily clinical practice remains
Source: Eur J Hosp Pharm. 2019 Nov 11;28(6):306–12. doi: 10.1136/ejhpharm-2019-001977 (PMC8552141; doi:10.1136/ejhpharm-2019-001977)
Supplement: Supplementary data [file ejhpharm-2019-001977supp001.pdf]

Appendix 1

List of drug substances included in the paediatric compounding survey across Dutch paediatric hospitals

Acenocoumarol	Flecainide (acetate)	Potassium citrate
Acetazolamide	Fludrocortisone	Potassium iodide
Acetylsalicylic acid	Folic acid	Potassium-sodium phosphate
Alimemazine	Folinic acid	Prednisolone
Allopurinol	Furosemide	Procarbazine
Amiodarone (hydrochloride)	Gabapentin	Propafenon
Amlodipine	Glycopyrronium	Propranolol
Ammonium chloride	Granisetron	Propylthiouracil
Aripiprazole	Hydrochlorothiazide	Pyrazinamide
Ascorbic acid	Hydrocortisone	Pyridoxine
Atenolol	Hydroxychloroquine	Pyrimethamine
Azathioprine	Imatinib	Ranitidine
Baclofen	Indomethacin	Retinol
Biotin	Isoniazid	Ribavirin
Biperiden	Isosorbide	Riboflavin
Bosentan	Labetalol	Sevelamer
Bumetanide	Lamotrigine	Sildenafil
Calcitriol	L-Arginine	Simvastatin
Calcium acetate	L-Citrulline	Sodium benzoate
Captopril	Levodopa + carbidopa 10: 1	Sodium chloride
Carglumic acid	Levofloxacin	Sodium selenite
Carvedilol	Linezolid	Sodium sodium bicarbonate
Chloral hydrate	Lisinopril	Sotalol (hydrochloride)
Chloroquine	Lorazepam	Spironolactone
Chlortalidon	Magnesium chloride	Sulfadiazine
Clobazam	Magnesium citrate	Sulfasalazine
Clonidine	Mefloquine	Tacrolimus
Codeine	Melatonin	Temazepam
Coffeine	Mercaptoethane sulfonic acid	Tetrahydrobiopterin
Colestyramine	Mercaptopurine	Theophylline
Cyclophosphamide	Methadone (hydrochloride)	Thiamazole
Dantrolene orally	Methotrexate	Thiamine
Dexamethasone	Metoprolol	Tioguanine
Dexamphetamine	Metoprolol (tartrate)	Tiopronine
Diazepam	Midazolam	Tocopherol acetate DL-alpha
Diazoxide	Naproxen	Tolterodine
Diclofenac	Nifedipine	Topiramate
Disodium hydrogen phosphate	Nilotinib	Tranexamic acid
Doxapram	Nitrazepam	Triamtereen
Enalapril	Nitrofurantoin	Trimethoprim
Esketamine	Ofloxacin (hydrochloride)	Ursodeoxycholic acid
Esomeprazole	Omeprazole	Valaciclovir
Ethambutol	Penicillamine	Valganciclovir
Etoposide	Perampanel	Vancomycin
Fenobarbital	Phenytoin	Zonisamide
Fenprocoumon	Phytomenadione	
Ferrochloride	Potassium chloride	
